# Late Researches in Epilepsy

**Published:** 1894-05

**Authors:** M. F. Clausius

**Affiliations:** First Assistant at the Northern Hospital for the Insane, Winnebago, Wis.


### LATE RESEARCHES IN EPILEPSY.

By PROF. DR. SEELIGMÜLLER.

(Translated for the JOURNAL from the *Centralblatt für die Gesammte Therapie*, March, 1894.)

By M. F. CLAUSIUS, M. D.,
First Assistant at the Northern Hospital for the Insane, Winnebago, Wis.

IN SPITE of the great number of remedies recommended for the treatment of epilepsy, the salts of bromine still remain the most reliable. Gauster comprises his experience in the treatment of epilepsy with the bromides in the following sentences: "The treatment of epilepsy with the bromides is at present by all means the most satisfactory, in the various forms and especially in idiopathic epilepsy." They must, as a rule, be taken for years.

The size of the dose must be regulated according to the individual by careful experiments and observations; either in regard to the increase or decrease of the dose, after a marked improvement has taken place. Under careful observation of the condition of the patient the dose may be increased to twenty grams a day, and this amount may be given for some time without developing dangerous symptoms.

The increase of dose is to be discontinued, or gradually diminished, or a different remedy is to be given temporarily or permanently: (*a*) if digestion is largely impaired and nutrition diminished; (*b*) if apex catarrh with slight dulness are developed; (*c*) if deep ulcerations of the skin are present.

Retardation of intelligence and development of mental stupor are generally no cause for discontinuance of the treatment with bromides, neither do they call for a decrease of the dose. Pulmonary tuberculosis, severe chronic diseases of the skin, and profound impairment of nutrition, contra-indicate the use of the bromides, providing the epileptic attacks do not directly endanger the life of the patient. A slight loss of flesh is no contra-indication, since an increase of weight may take place during the continuance of the bromide treatment. During the bromide treatment a nourishing diet must be given. From time to time the condition of the lungs and the skin should be examined.

Féré gives the following directions for the use of the bromides in epilepsy : the preparation must be chemically pure and especially be free from admixture with the salts of iodine, chloride of sodium and sulphate and carbonate of potassium. Since the bromides, when taken into an empty stomach, are liable to cause gastralgia, it is best to administer them at the beginning of the meal. They are liable to cause dental caries, and, therefore, the teeth and mouth must be kept clean. Success can only be expected when the physiological action is manifested—namely, a sense of weariness, sleepiness, anaphrodisia and suspension of nausea by means of mechanical irritation of the pharynx and base of the tongue. This last named sign is, to a certain extent, the criterion of the saturated condition, the exceeding of which is unnecessary, but which it is necessary to maintain until certain of a cure, or, better said, as a cure is never assured, as Voisin expresses himself, bromide of potassium should be constantly given as a nourishment to the cured epileptic.

If the attacks have ceased for one or two years, we may then try to gradually discontinue the bromides, as, for instance, give them every third, or every other, or for only eight days each month. It is well to be sure in each case that the pharynx reflexes are absent. The bromide treatment should never be stopped abruptly, as in such cases nearly always a severe attack occurs within nine days, which may endanger the life of the patient; even when no attack occurs within a short time, a fatal attack may occur even after several months (Le Grand de Saulle). In regard to the dose, it might be said that in place of continuing a given dose from day to day, Charcot recommends to begin with increasing doses, by raising the dose each week by one gram. For instance, to increase it from four to seven grams within four weeks and then again to decrease it within the same time to four grams. In England, a large dose is given every three or four days—15, 20 to 25 grams at meal times. Puche has witnessed 40

grams given in one day without any ill effects. Féré has, however, observed bromism after doses of only 20 grams. Further observations will have to be awaited.

The bromides act favorably in all forms of epilepsy, either in the psychical form, or in those accompanied with pain, migraine, or migraine ophthalmique, and, finally, in those cases that are complicated with external and internal spasms (spasmus glottidis). To prevent eruptions of the skin while taking bromides, rigid cleanliness of the skin and especially frequent bathings are to be recommended. Against the occasionally occurring salivation, tannic acid and hyoscyamus are efficacious. To prevent real ulcerations of the skin, which are apt to appear when very large doses, about 14 grams per day, are given, Féré recommends a method which he calls intestinal antisepsis. He gives naphthol in 4 gram, and salicylate of bismuth in 2 gram doses. With this treatment the digestive disturbances resulting as consequences of large doses of bromides are prevented. This medication is well tolerated for months.

To prevent the general evil actions of the bromide preparations, free diuresis is to be maintained, eventually by diuretics. If bromine poisoned epileptics are attacked by an infectious disease, an adynamic and typhoid condition soon appears. The use of bromides during pregnancy has no ill effect upon the fetus, but is likely to leave a salutary influence ; besides, the bromides should show a prophylactic action, if puerperal eclampsia has been present in previous pregnancies. They also act favorably in pronounced eclampsia, as well as in status epilepticus. It is well to consider the warning of Savage in regard to the careless use of the bromides, as he cites a case that came especially under his notice, in which the psychical condition became rapidly worse, seemingly as a consequence of the suppression of the epileptic attacks by the use of the bromides. Against the psychical symptoms of bromide poisoning, Wildermuth recommends, besides the cessation of the bromides, black coffee, rain douches on the back in warm baths, and massage of the extremities. Besides the usually employed bromide salts, such as potassium, sodium and ammonium, a great many other bromide combinations have been tried with more or less success. Next to the above mentioned is the bromide of lithium. Of late, Féré recommended bromide of strontium in the same small doses as the potassium salt. Confirmation of success by others must be awaited.

The observation of Doyon, who found deposits of bromides in the organs of men and animals who had taken the salts for some time, is interesting. He found them more frequently existing in the liver than in the brain. The bromide of nickel, especially recommended for headache and convulsions by Da Costa, was tried by Bourneville on eighteen patients, of which seven were idiopathic and eleven symptomatic epilepsy, but proved efficacious only in a single case, inasmuch as the attacks had nearly ceased, which result had lasted for three years. In two other cases a very slight improvement was manifested. The other fifteen were even aggravated. The usual dose is 3 decigrams, and the maximum dose 6 decigrams. Stomach difficulties are readily brought on. The bromide of camphor is said to be quite efficacious in the vertiginose form of epilepsy. Bromide of arsenic is recommended by Clement; bromide of calcium, in doses of from 5 decigrams to 2 grams, by Hammond; bromide of zinc, in the same dose, by Charcot, Rochefontaine, Bourneville; bromide of gold, in doses of 8 milligrams, by Bourneville and Goubart. All three have been tried, occasionally with favorable effect, but they are by no means as certain as the commonly used bromides. The bromide of ethel, known as an anesthetic, has been tried by inhalation several times a day (d'Ollier and Bourneville), apparently without special influence on the course of the disease. The ethelin bromide, however, according to observation by Donath, on twenty-one patients, acted similarly to the commonly used bromides. Donath recommends the following formula:

| | | |
|---|---|---|
| ℞. | Ethelin bromide.................. | 5, 0 |
| | Emuls. oleos..................... | 100, 0 |
| | Ol. menth. pip., gtt............... | 2, 0 |
| M. | | |

Of this mixture adults take thirty drops, three times a day, in one-half glass of sweetened water, adding ten drops every third day until seventy drops are reached, equal to one teaspoonful. Children, eight to ten years of age, commence with from ten to twenty drops. In case the stomach does not tolerate this mixture, which, however, seldom occurs, a few drops of tincture opium may be added. Exanthema never appeared on giving these small doses. This remedy can also be prescribed in gelatine capsules, each containing three drops, with six drops of oil of sweet almonds, and from two to four capsules may be given two or three times a day. Donath observed, besides convulsions, muscular twitching in the extremities with retained consciousness with this treatment in three patients, which he interpreted abortive attacks. The rubidium-ammonium bromide was first recommended by Laufenauer as an anti-epileptic, and tried in seventeen cases. In six of these it proved more efficacious than the bromide of potassium. On account of the great price of this preparation—$20 per pound—and a single dose being from two to five, and the daily dose from seven to eight grams, extensive experiments can hardly be made at present. Rottenbiller has treated five epileptics with it, and has given them a daily dose of six grams. He observed a remarkable diminution of the attacks. This, however, was not lasting, and after the discontinuance of the drug the attacks again occurred with the usual frequency. Hyperosmic acid, in pill-form, daily dose five milligrams, gradually increased to from ten to fifteen milligrams, has been tried by Schroeder, on ten epileptics, without success, excepting in one case in which the attacks were influenced. Borax, first recommended by Gowers, Folsom and others, has of late been tried by Féré and Lamy and also by Stewart. The daily dose is from three to six grams. Sherrington calls attention to the favorable influence on the night attacks and, therefore, advises to administer the bromides also in cases where attacks occur in the day time. Gowers, who advised to commence with a daily dose of one gram, gradually increased to six grams, has called attention to two deleterious effects produced by borax—namely, diarrhea and psoriasis.

Féré, who treated twenty-two epileptics with doses varying from 1 to 3 grams, has observed momentary improvement only in three of these cases, and also mentions that the patients complained of nausea and diarrhea. Besides this, there also appeared eczema on the lateral surfaces of the body and on the arms of two of the patients, which did not heal until six weeks after the discontinuance of borax. Sclerotic acid, commenced with daily doses of 10 centigrams, and gradually increased to from 25 to 30 centigrams, may also be administered subcutaneously in doses of from 15 to 36 milligrams, maximum dose, 6 centigrams, was tried by Bourneville and Bricon on twelve epileptics, and in five of them produced an insignificant decrease of attacks, but in some an immediate decrease of bodily weight was observed. Curare, first recommended by Kunze, and later on by Edlefsen, was tried by them on twenty-three patients with so little effect, that they recommended to have this not indifferent remedy discarded from the list of anti-epileptics. The firm of Christy & Co., of London, prepared a tincture of simalo, from a South American fruit belonging to the family ysope (the *capparis corriacea*), which is highly recommended in Peru and Bolivia as an anti-spasmodic. This tincture was first administered by Hale White, in doses of from 3 to 5 grams, three times daily, to seven patients, with encouraging success. Eulenburg, who treated four epileptics with this tincture, commencing with doses of one-half teaspoonful, gradually increased to 2 teaspoonfuls, two or three times a day, as an experiment, found it not wholly without effect, but not equal to the bromide preparations. Osler's experiments with nitro-glycerine have been accompanied with but little success, and in two cases the remedy had to be discarded on account of ill effects, such as headache and vertigo. It may be said that very little effect has been produced in the treatment of epilepsy with antifebrin, in doses of from 25 centigrams to 2 grams per day. Neither have experiments with antipyrin proved successful. Lemoine, however, has seen success with antipyrin, firstly, where the attacks occurred at the time of menstruation; secondly, in epilepsia larvata; and, thirdly, when complicated with migraine. A combination of bromide of potassium with antipyrin might prove successful. Amadei claims to have produced a cessation of the attacks for four months with the above mixture. Finally, amylenhydrate, first recommended by Wildermuth, has not fulfilled the expectations of later experiments. The high price of the drug must also be considered. Naecke, however, observed a marked decrease of attacks in old cases of epilepsy, often in a remarkable degree. Later, in another series of observations, with thirty epileptic men, he observed an increase of the attacks and much mental dulness, after a use of this remedy for several weeks. Babes injected the brain and spinal marrow of sheep, from five to six times a week, and obtained better results than with any other remedy recommended for epilepsy. Wildermuth recommends wet packings in cases of status epilepticus, and large doses of bromide of potassium, given either per clysma or hypodermically. Three times the amount of the daily dose should be given if the same is not too large. For cardiac weakness, large doses of camphor should be administered subcutaneously. He found nitrate of amyl inefficacious, and chloroform proved useful in only a single case.

